# Management of distraction injury of the lumbosacral junction with unilateral perched facet

**DOI:** 10.4103/2152-7806.77278

**Published:** 2011-03-03

**Authors:** Clemens M. Schirmer, Erica F. Bisson

**Affiliations:** 1 Division of Neurosurgery, Baystate Medical Center and Tufts University School of Medicine, Springfield, Massachusetts, USA; 2 Department of Neurosurgery, Clinical Neurosciences Center, University of Utah, Salt Lake City, Utah, USA

**Keywords:** Facet dislocation, operative management, trauma

## Abstract

**Background:**

Traumatic unilateral facet dislocation without fracture is an uncommon injury of the lumbosacral junction. We describe a case of a unilateral perched L5–S1 facet causing axial back pain and radiculopathy provoked by motion.

**Case Description:**

The patient underwent reduction with complete facetectomy followed by internal fixation at L5–S1, facilitating decompression of the S1 nerve root. Postoperatively, the patient reported improvement in her pain.

**Conclusions:**

This injury can be recognized using subtle clues, such as transverse process fractures and/or widened posterior elements. Despite its rarity, when identified, this injury can be characterized using the new TLICS system for thoracolumbar fractures and should be managed accordingly.

## INTRODUCTION

Traumatic unilateral or bilateral facet dislocations without fracture of the cervical spine are common injuries, indicative of injury of the stabilizing ligaments, and are routinely managed with reduction and operative fixation. In contrast, facet dislocations without fracture of the lumbar spine are rare. The superior articular process may be partially or completely dislocated, and the dislocations may involve either one or both articular processes.[49] Most reported cases of this injury pattern localize to the lumbosacral junction and have been managed both conservatively and, more recently, with open reduction and fusion.[1,2,5,8,9,11] The Thoracolumbar Injury Classification and Severity (TLICS) score for thoracolumbar fractures can provide guidance.[44,45] We describe a case of a unilateral perched L5–S1 facet causing axial back pain and radiculopathy provoked by motion, discuss the literature, and explain the rationale for treatment based on the TLICS classification.

## CASE DESCRIPTION

**Figure 1:**
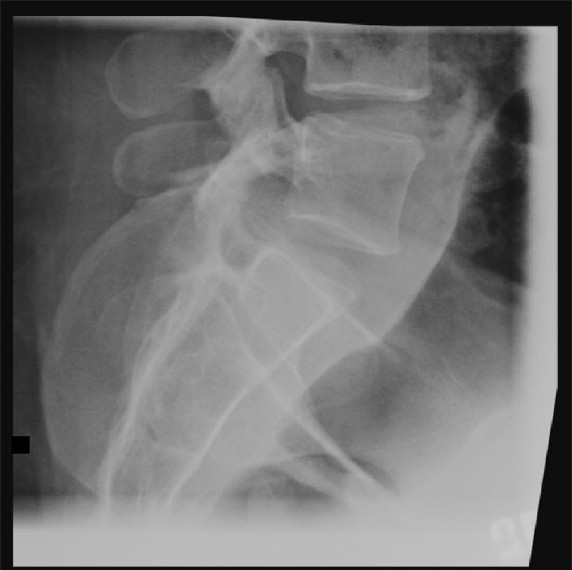
Plain lateral radiograph of the lumbar spine, demonstrating subtle widening of one of the L5–S1 neuroforamina

**Figure 2:**
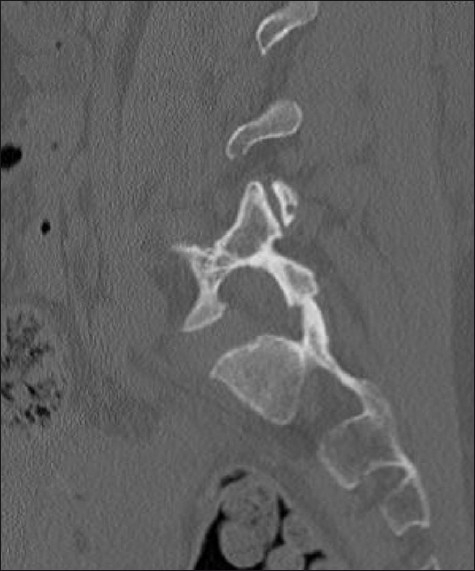
Computed tomography of the lumbar spine. Sagittal reconstruction through the perched right L5–S1 facet joint

**Figure 3:**
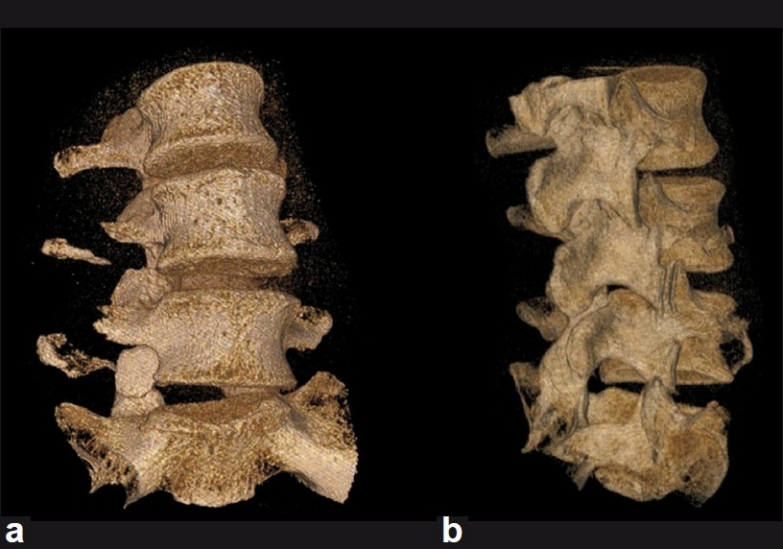
Anterior (a) and right lateral (b) views of the three-dimensional reconstruction of the computed tomography scan of the lumbar spine, demonstrating the unilateral perched facet joint and asymmetric widening of the L5–S1 disc space

**Figure 4:**
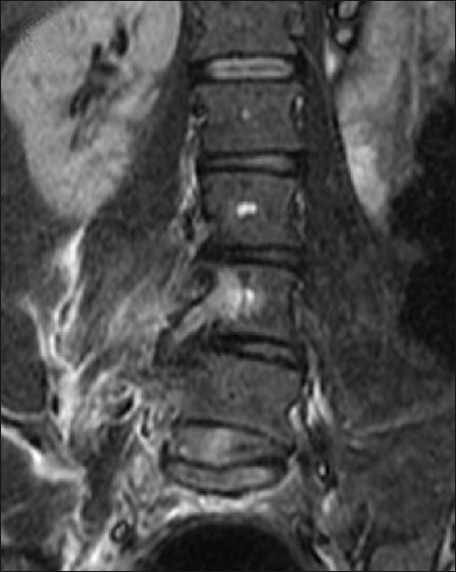
Coronal inversion recovery (STIR) weighted image showing edema and asymmetric widening of the L5–S1 disc space

A 46-year-old woman presented in transfer from a community hospital after sustaining injuries from a motor-vehicle accident in which she was a restrained driver. During evaluation at the community hospital, no gross signs of internal or external injuries and no neurologic deficit were noted; however, the patient complained of significant pain and spasms in the lower back, worsened by an upright position. She also complained of right leg pain with a sharp quality when she would change position. Lumbar radiographs showed a potential widening of one of the L5–S1 neuroforamina [Figure 1]. Evaluation with thin-slice computed tomography (CT) with coronal and sagittal plane reconstructions demonstrated a unilateral dislocation of the right L5–S1 facet joint with a perched facet [Figure 2]. Fractures with significant diastasis of the fragments of the right L4 and L5 transverse processes were noted (not shown). Magnetic resonance (MR) imaging of the lumbar spine did not show significant disruption of the intervertebral disc; however, unilateral distraction of the disc space is visible on the CT and MR images [Figures 3 and 4]. We calculated the patient’s TLICS score to be 8, 3 points for translational/rotational injury morphology, 3 points for injured posterior ligamentous complex, and 2 points for radiculopathy.

**Figure 5:**
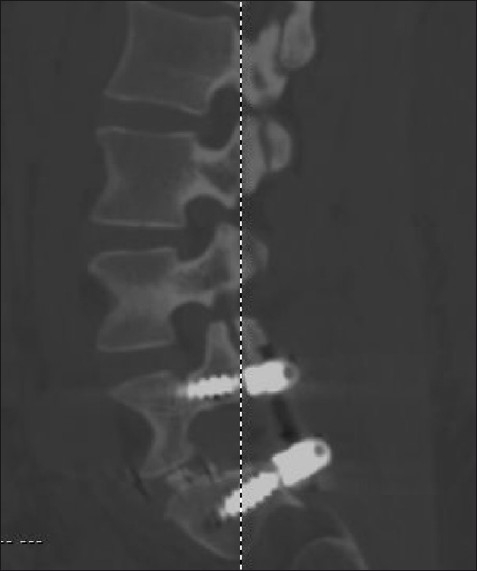
Sagittal reconstruction of the postoperative computed tomography showing the extent of resection of the articular processes and the transpedicular posterior segmental fixation on the right side. A portion of the polyetheretherketone (PEEK) interbody graft is visible

**Figure 6:**
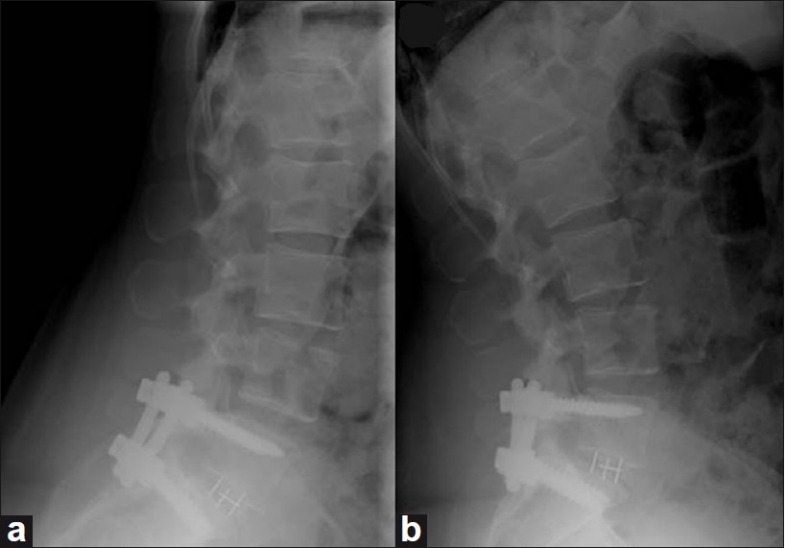
Lateral flexion (a) and extension (b) radiographs of the lumbar spine demonstrating pedicle screw instrumentation and interbody graft at L5–S1 with no significant motion between flexion and extension at the instrumented level

Surgical intervention was undertaken because of the presence of clinical instability and her TLICS score. We found obvious disruption of the posterior tension band, including the interspinous ligaments between L4, L5, and S1 with associated soft tissue hematoma. After subperiosteal dissection, the naked superior articular process of S1 was visible, with the most caudal aspect of the inferior articular process of L5 lying cephalad and ventral to it, causing a clockwise rotation of L5. Reduction was accomplished with a complete facetectomy on the right followed by internal fixation with interbody graft and pedicle screw instrumentation at the L5–S1 level, facilitating complete decompression of the S1 nerve root [Figure 5]. Postoperatively, the patient reported immediate and sustained improvement in her back and leg pain. Dynamic X-rays obtained at 6 months showed no significant motion at the operated level [Figure 6].

## DISCUSSION

Trauma to the lumbar spine typically results in injuries at the thoracolumbar junction. In a large series more than half of all fractures involved T12 or L1.[27] Approximately 60% were classified as compression or burst fractures resulting most often from a flexion–compression-type injury.

The diagnosis of lumbosacral dislocation may be missed because radiographs taken as part of a trauma evaluation may be inadequate to visualize an abnormal relationship of the lumbosacral facets. The presence of transverse process fractures, as seen in our case, should alert the medical team to the possibility of a more serious injury.[23,53,54] Thin-sliced CT images with sagittal and coronal reconstructions demonstrate the injury with clarity. Additionally, we found three-dimensional reconstructions particularly helpful to discern the relationship of the L5 and S1 articular processes.

For subacute dislocation, surgical treatment is complex. The reported cases treated conservatively had excellent clinical outcomes.[5,23,31,54] In contrast, the conservative treatment of acute fracture–dislocation of L5–S1 is likely ineffective because of significant instability, necessitating open reduction and internal fixation.[31]

Although our patient was neurologically intact without canal compromise, with a score of 8 on the TLICS system, optimal management of this lesion is operative reduction and fixation.[44,45] Additionally, we considered this lesion unstable according to the comprehensive definition by White and Panjabi.[52]

Lumbosacral dislocations are usually associated with high-energy trauma, and patients often suffer from associated visceral lesions, extraspinal fractures, and secondary spine fractures, including transverse process fractures in the majority.[6] Our patient suffered 2 transverse process fractures without evidence of visceral injury.

In a review of the literature on lumbosacral dislocations, we found 93 cases that were previously reported [Table 1].[1–26,28,30–43,46,48–51,53,54] Purely ligamentous injury akin to the findings in our case were less prevalent and have been reported in only a few cases.[2,42,48] Although a few cases were managed conservatively, most patients underwent open reduction and fusion, by variable approaches and fusion techniques. Primary facet dislocations involving the lumbosacral spine are rare and have been reported to occur mainly at the lumbosacral junction in association with anterior subluxation.[1,2,5,8,9,11] A case of lateral subluxation associated with a unilateral locked facet in the lumbar spine has also been reported.[29]

**Table 1:** Review of the existing literature concerned with the management of lumbosacral dislocations

Report	Number of cases	Treatment	Dislocation type	Cause
Dewey *et al,*1968[16]	2			Trauma
Samberg, 1975[39]	1			Trauma
Fardon, 1976[19]	1	Surgery		Trauma
Newell, 1977[31]	1	Conservative		Trauma
Jackson *et al,*1979[25]	3	Surgery		Trauma
Zoltan *et al,*1979[54]	1		Unilateral	
Griffin *et al,*1980[21]	1	Conservative		
Das De, 1981[14]	4	Surgery		
Morris, 1981[30]	1		Unilateral	
Boger *et al,*1983[5]	1		Unilateral	
Nicholson, 1983[32]	1	Surgery	Unilateral	
Herron *et al,*1984[22]	1	Surgery		
Boyd *et al,*1985[7]	1	Conservative		
Resnik *et al,*1985[36]	1			
Wilchinsky, 1987[53]	1			
Grabe, 1988[20]	1			Tonic-clonic seizure
Miz *et al,*1988[28]	1		Unilateral	
Cohn *et al,*1989[10]	1		Bilateral	Trauma
Kramer *et al,*1989[26]	1		Unilateral	
Carl *et al,*1991[8]	1		Unilateral	
Connolly *et al,*1992[11]	4	Surgery	Unilateral	
Pellise *et al,*1992[33]	1	Surgery	Unilateral	Trauma
Van Savage *et al,*1992[46]	1	Surgery	Unilateral	
Barquet *et al,*1993[3]	1		Unilateral	Trauma
Davis *et al,*1993[15]	1	Surgery	Bilateral	Trauma
Beguiristain *et al,*1995[4]	1	Conservative	Bilateral	Trauma
Hilibrand *et al,*1995[23]	4	Surgery	Bilateral	Trauma
Fabris *et al,*1996[18]	12	Surgery	Bilateral	Pediatric, degenerative
Steinitz *et al,*1997[40]	1	Surgery	Bilateral	Trauma, delayed
Aihara *et al,*1998[1]	7	Surgery	Both	Trauma
Roche *et al,*1998[37]	1	Surgery	Bilateral	Trauma
Carlson *et al,*1999[9]	2	Surgery		Trauma
Fabris *et al,*1999[17]	3	Surgery		Trauma
Hodges *et al,*1999[24]	1	Surgery	Bilateral	Trauma
Verlaan *et al,*2001[48]	1	Surgery	Bilateral	Trauma
Cruz-Conde *et al,*2003[12]	1	Surgery	Bilateral	Trauma
Arnold *et al,*2004[2]	1	Surgery	Bilateral	Trauma
Boldin *et al,*2004[6]	1		Unilateral	Trauma
Stuart *et al,*2004[41]	1		Unilateral	Trauma
Tsirikos *et al,*2004[43]	2	Surgery	Bilateral	Trauma, delayed
Vialle *et al,*2004[51]	4	Surgery		Trauma
Vialle *et al,*2005[50]	1	Surgery	Unilateral	Trauma
Reinhold *et al,*2006[35]	1	Surgery	Bilateral	Trauma
Saiki *et al,*2006[38]	1	Surgery	Bilateral	Trauma
Vialle *et al,*2007[49]	11	Surgery	Both	Trauma
Reddy *et al,*2008[34]	2	Surgery	Unilateral	Trauma
Szentirmai *et al,*2008[42]	1		Unilateral	Trauma
Daniels *et al,*2009[13]	1	Surgery	Bilateral	Trauma

In young children, this injury has been managed successfully by cast immobilization[23]; however, conservative treatment of fracture–dislocation of L5 in adolescents is generally ineffective because the lesion is considered fundamentally unstable as a result of the incurred severe bone and ligamentous damage.[6]

Unlike the lumbar facets with a relative sagittal orientation, the L5–S1 facet has a more coronal alignment.[47] Thus, the lumbosacral junction behaves similar to the cervical spine, and a unilateral perched facet implies a rotatory component.

The management of these rare injuries is still controversial. Boldin and coworkers[6] argued that open reduction and internal fixation are indicated for the management of acute lumbosacral dislocation in all but children. For open reduction, the fifth lumbar and first sacral articular processes are distracted by temporarily increasing the flexion deformity. Occasionally, disimpaction and reduction can be achieved only after partial excision of the tip of the superior facet of S1.[8,11,28] In cases of intervertebral disc derangement, anterior interbody fusion has been advocated.[1] We chose operative reduction and fixation for our patient based on the clinical instability demonstrated by severe back and radicular pain associated with motion, which resulted in improvement of her symptoms.

## CONCLUSIONS

A unilateral lumbosacral facet dislocation is a rare injury that has been managed both conservatively and surgically. Transverse process fractures may point to the diagnosis of this injury, which otherwise may be missed on routine evaluation. Despite its rarity, this injury pattern fits into the categories of the Thoracolumbar Injury Classification and Severity Score (TLICS) system for thoracolumbar fractures and can be managed accordingly. Open reduction and fixation is safe and efficacious.
