# Supplementary material for: Management of distraction injury of the lumbosacral junction with unilateral perched facet
Source: Surg Neurol Int. 2011 Mar 3;2:26. doi: 10.4103/2152-7806.77278 (PMC3050071; doi:10.4103/2152-7806.77278)
Supplement: Related texts

# Article commentary: Commentary

**Authors:** Ron Pawl

**Affiliations:** Department of Neurosurgery, University of Illinois, Chicago 900 Westmoreland, Suite LL50, Lake Forest, Chicago, IL, USA. E-mail: ron@pawl.com

This report alerts neurosurgeons to the need for careful analysis of diagnostic radiographs when a patient has been involved in a potentially high-impact injury to the spine. The authors point out that transverse process fractures, in part, directed them to the unilateral dislocation at the lumbosacral junction. However, such dislocations may occur without other fractures[1,2] and thus fail to give a clue to such an injury at that location, further underlining the need for a careful radiographic analysis in lumbar spine trauma cases. The first report cited above also documents forced lateral flexion-extension as the major factor in the traumatic mechanism.

The authors of this report made use of the Thoracolumbar Injury Classification and Severity Scale – TLICSS, originally proposed by The Spine Trauma Study Group as reported by Vaccaro *et al*. in 2005, in helping to make the decision to surgically treat this dislocation. Spine surgeons should familiarize themselves with this classification, which has been verified in a number of reports[3–5] and which can be found readily online using the Google search engine.

## References

[55] Krbec M, Hrabeta P (2010). Unilateral isolated dislocation at the lumbosacral junction: Lateral flexion-distraction as a major vector in the mechanism of injury: Case report and trauma mechanism analysis. Eur Spine J.

[56] Blecher R, Geftler A, Anekstein Y, Mirovsky Y (2010). Isolated unilateral facet dislocation of the lumbosacral junction. J Bone Joint Surg Br.

[57] Koh YD, Kim DJ, Koh YW (2010). Reliability and Validity of Thoracolumbar Injury Classification and Severity Score (TLICS). Asian Spine J.

[58] Patel AA, Vaccaro AR (2010). Thoracolumbar spine trauma classification. J Am Acad Orthop Surg.

[59] Lenarz CJ, Place HM (2010). Evaluation of a new spine classification system, does it accurately predict treatment?. J Spinal Disord Tech.
